# Supplementary material for: Prevalence of Myocardial Injury and Myocardial Infarction in Patients with a Hypertensive Emergency: A Systematic Review
Source: Diagnostics (Basel). 2022 Dec 26;13(1):60. doi: 10.3390/diagnostics13010060 (PMC9818542; doi:10.3390/diagnostics13010060)
Supplement: Supplementary file 1 [file diagnostics-13-00060-s001.zip › diagnostics-2010232-supplementary.pdf]

Table S1. Detail of database search strategy.

Database	Search terms/strategy
PubMed	(("malignant hypertension"[All Fields] OR "hypertensive emergency"[All Fields] OR "hypertensive emergencies"[All Fields] OR "hypertensive crisis"[All Fields] OR "hypertensive crises"[All Fields] OR "acute hypertensive crisis"[All Fields] OR "acute hypertensive crises"[All Fields] OR "accelerated hypertension"[All Fields]) AND ((("myocardial ischemia"[All Fields] OR "unstable angina"[All Fields] OR "raised cardiac troponin"[All Fields] OR "raised troponin I"[All Fields] OR "raised troponin T"[All Fields] OR "raised cardiac enzymes"[All Fields] OR "elevated cardiac troponin"[All Fields] OR "elevated troponin I"[All Fields] OR "elevated troponin T"[All Fields] OR "elevated cardiac enzymes"[All Fields] OR "acute coronary syndrome"[All Fields] OR "acute myocardial infarction"[All Fields]) AND "ST-Elevation myocardial infarction"[All Fields]) OR "Non-ST elevation myocardial infarction"[All Fields] OR "myocardial infarction"[All Fields] OR "heart attack"[All Fields] OR "pulmonary edema"[All Fields])) AND (humans[Filter])
Web of Science	("myocardial ischemia" OR "unstable angina" OR "raised cardiac troponin" OR "raised troponin I" OR "raised troponin T" OR "raised cardiac enzymes" OR "elevated cardiac troponin" OR "elevated troponin I" OR "elevated troponin T" OR "elevated cardiac enzymes" OR "acute coronary syndrome" OR "acute myocardial infarction" "ST-Elevation myocardial infarction" OR "Non-ST elevation myocardial infarction" OR "myocardial infarction" OR "heart attack" OR "pulmonary edema")<br>AND<br>("malignant hypertension" OR "hypertensive emergency" OR "hypertensive emergencies" OR "hypertensive crisis" OR "hypertensive crises" OR "acute hypertensive crisis" OR "acute hypertensive crises" OR "accelerated hypertension")
EBSCOHost	TX ((("myocardial ischemia" OR "unstable angina" OR "raised cardiac troponin" OR "raised troponin I" OR "raised troponin T" OR "raised cardiac enzymes" OR "elevated cardiac troponin" OR "elevated troponin I" OR "elevated troponin T" OR "elevated cardiac enzymes" OR "acute coronary syndrome" OR "acute myocardial infarction" "ST-Elevation myocardial infarction" OR "Non-ST elevation myocardial infarction" OR "myocardial infarction" OR "heart attack" OR "pulmonary edema"))) AND TX (("malignant hypertension" OR "hypertensive emergency" OR "hypertensive emergencies" OR "hypertensive crisis" OR "hypertensive crises" OR "acute hypertensive crisis" OR "acute hypertensive crises" OR "accelerated hypertension"))

Table S2. Studies excluded from the review.

	Reference	Reason for exclusion
1	Afonso, L., Bandaru, H., Rathod, A., Badheka, A., Ali Kizilbash, M., Zmily, H., ... & Weaver, W. D. (2011). Prevalence, determinants, and clinical significance of cardiac troponin-I elevation in individuals admitted for a hypertensive emergency. *The Journal of Clinical Hypertension*, *13*(8), 551-556.	No information on prevalence of target organ damage or myocardial injury
2	Rashed Al Bannay, M. D., CBIS, M. U., & Aysha Husain, M. D. (2018). Clinical characteristics of acute heart failure patients. *Bahrain Medical Bulletin*, *40*(1)	No information of other acute target organ damage or cardiac troponin
3	Alhasan, D., & Yaseen, A. (2021). Hypertensive Emergencies in the Ambulance: Characteristics, Clinical Presentations and Complications–A Prospective Cohort Study. *Open Access Emergency Medicine: OAEM*, *13*, 75.	Out of hospital cohorts.
4	Shantsila, A., Shantsila, E., Beevers, D. G., & Lip, G. Y. (2017). Predictors of 5-year outcomes in malignant phase hypertension: the West Birmingham Malignant Hypertension Registry. *Journal of hypertension*, *35*(11), 2310-2314.	No report of acute hypertension target organ damage
5	Astarita, A., Covella, M., Vallelonga, F., Cesareo, M., Totaro, S., Ventre, L., ... & Milan, A. (2020). Hypertensive emergencies and urgencies in emergency departments: a systematic review and meta-analysis. *Journal of hypertension*, *38*(7), 1203-1210.	Review
6	Berger, M., Emir, M., Brünnler, T., Rockmann, F., & Lehmann, R. (2018). Non-coronary predictors of elevated high-sensitive cardiac troponin T (hs-cTnT) levels in an unselected emergency patient cohort. *Clinical Cardiology*, *41*(8), 1055-1061.	No details of target organ injury
7	Boone, S., & Kuo, D. (2018). Novel Biomarkers to Detect Target Organ Damage in Acute Hypertension. *Current Hypertension Reports*, *20*(3), 1-8.	Review
8	Chaulin, A. (2021). Clinical and diagnostic value of highly sensitive cardiac troponins in arterial hypertension. *Vascular Health and Risk Management*, *17*, 431.	Study not targeted at hypertensive emergency. No report on target organ damage
9	Elsharnoby, H. R., Bhogal, J., Palatnic, L., Elsheikh, E., Khalil, M., Kayani, W., & Maraey, A. M. (2021). Type 2 Myocardial Infarction in Young Adults: Insights From the National Readmission Database. *Cureus*, *13*(11).	No report on acute hypertension mediated organ damage. Focus on type 2 myocardial infarction in young adults.

10	Gegenhuber, A., & Lenz, K. (2003). Hypertensive emergency and urgence. *Herz*, *28*(8), 717.	Review article
11	Guimarães, P. O., Leonardi, S., Huang, Z., Wallentin, L., Van de Werf, F., Aylward, P. E., ... & Tricoci, P. (2018). Clinical features and outcomes of patients with type 2 myocardial infarction: insights from the Thrombin Receptor Antagonist for Clinical Event Reduction in Acute Coronary Syndrome (TRACER) trial. *American Heart Journal*, *196*, 28-35.	Review article
12	Harrison, N., Pang, P., Collins, S., & Levy, P. (2021). Blood pressure reduction in hypertensive acute heart failure. *Current hypertension reports*, *23*(2), 1-11.	Review article
13	Hunter, B. R., Martindale, J., Abdel-Hafez, O., & Pang, P. S. (2017). Approach to acute heart failure in the emergency department. *Progress in cardiovascular diseases*, *60*(2), 178-186.	Review article
14	Kim, B. S., Kim, H. J., Lyu, M., Kim, W. D., Lee, Y., Kim, M., ... & Shin, J. H. (2021). Clinical characteristics, practice patterns, and outcomes of patients with acute severe hypertension visiting the emergency department. *Journal of hypertension*, *39*(12), 2506-2513	Similar cohort with reference number 12
15	Langlo, K. A. R., Silva, G. J. J., Overrein, T. S., Adams, V., Wisløff, U., Dalen, H., ... & Hallan, S. I. (2021). Circulating microRNAs May Serve as Biomarkers for Hypertensive Emergency End-Organ Injuries and Address Underlying Pathways in an Animal Model. *Frontiers in cardiovascular medicine*, 431.	Animal study
16	Mandi, D. G., Yaméogo, R. A., Sebgo, C., Bamouni, J., Naibé, D. T., Kologo, K. J., ... & Zabsonré, P. (2019, October). Hypertensive crises in sub-Saharan Africa: clinical profile and short-term outcome in the medical emergencies department of a national referral hospital in Burkina Faso. In *Annales de Cardiologie et d'Angéiologie* (Vol. 68, No. 4, pp. 269-274).	Conflicting report on prevalence of the different acute hypertension mediated organ damage, totalling 124%
17	Maraey, A., Elzanaty, A. M., Salem, M., Khalil, M., Elsharnoby, H., Younes, A., ... & Siragy, H. M. (2021). Relation of Type 2 Myocardial Infarction and Readmission With Type 1 Myocardial Infarction in Hypertensive Crises (from a Nationwide Analysis). *The American journal of cardiology*, *161*, 56-62.	No report on prevalence of acute hypertension mediated organ damage

18	Morsi, R. Z., Chehab, O., Kanj, A., & Abidov, A. (2021). Hypertensive Emergency in Heart Failure: Trends, Risk factors and Outcomes from a Nationwide Analysis 2005–2014. *High Blood Pressure & Cardiovascular Prevention*, *28*(6), 619-622.	Short communication.
19	Paini, A., Tarozzi, L., Bertacchini, F., Aggiusti, C., Rosei, C. A., De Ciuceis, C., ... & Muiesan, M. L. (2021). Cardiovascular prognosis in patients admitted to an emergency department with hypertensive emergencies and urgencies. *Journal of Hypertension*, *39*(12), 2514-2520.<br>Papadopoulos, D. P., Sanidas, E. A., Viniou, N. A., Gennimata, V., Chantziara, V., Barbetseas, I., & Makris, T. K. (2015). Cardiovascular hypertensive emergencies. *Current hypertension reports*, *17*(2), 1-6.	Outcome study. Similar cohort with reference number 15
20	Papadopoulos, D. P., Sanidas, E. A., Viniou, N. A., Gennimata, V., Chantziara, V., Barbetseas, I., & Makris, T. K. (2015). Cardiovascular hypertensive emergencies. *Current hypertension reports*, *17*(2), 1-6.	Review article
21	Papadopoulos, D. P., Mourouzis, I., Thomopoulos, C., Makris, T., & Papademetriou, V. (2010). Hypertension crisis. *Blood pressure*, *19*(6), 328-336.	Review article
22	Peacock, F., Amin, A., Granger, C. B., Pollack Jr, C. V., Levy, P., Nowak, R., ... & Stat Investigators. (2011). Hypertensive heart failure: patient characteristics, treatment, and outcomes. *The American journal of emergency medicine*, *29*(8), 855-862.	No report on acute hypertension mediated organ damage. Focus on hypertensive heart failure
23	Saladini, F., Mancusi, C., Bertacchini, F., Spannella, F., Maloberti, A., Giavarini, A., ... & Muiesan, M. L. (2020). Diagnosis and treatment of hypertensive emergencies and urgencies among Italian emergency and intensive care departments. Results from an Italian survey: Progetto GEAR (Gestione dell'Emergenza e urgenza in ARea critica). *European Journal of Internal Medicine*, *71*, 50-56.	No report on acute hypertension mediated organ damage
24	Salkic, S., Batic-Mujanovic, O., Ljuca, F., & Brkic, S. (2014). Clinical presentation of hypertensive crises in emergency medical services. *Materia socio-medica*, *26*(1), 12.	No report on acute hypertension mediated organ damage
25	Shao, P. J., Sawe, H. R., Murray, B. L., Mfinanga, J. A., Mwafongo, V., & Runyon, M. S. (2018). Profile of patients with hypertensive urgency and	No report of myocardial infarction or myocardial injury

	emergency presenting to an urban emergency department of a tertiary referral hospital in Tanzania. *BMC cardiovascular disorders*, *18*(1), 1-7.	
26	Sharma, B. K., Singh, G., & Sagar, S. (1994). Malignant Hypertension in North West India A Hospital Based Study. *Japanese heart journal*, *35*(5), 601-609.	Focused on malignant hypertension. No report on acute hypertension mediated organ damage
27	Slama, M., & Modeliar, S. S. (2006). Hypertension in the intensive care unit. *Current Opinion in Cardiology*, *21*(4), 279-287.	Review article
28	Thiele, S., Britz, S., Landsiedel, L., Wallaschofski, H., & Lohmann, T. (2008). Short-term changes in hsCRP and NT-proBNP levels in hypertensive emergencies. *Hormone and metabolic research*, *40*(08), 561-565.	No report on acute hypertension mediated organ damage
29	Wildi, K., Twerenbold, R., & Mueller, C. (2015). How acute changes in cardiac troponin concentrations help to handle the challenges posed by troponin elevations in non-ACS-patients. *Clinical biochemistry*, *48*(4-5), 218-222.	Review article
30	Nkoke, C., Noubiap, J. J., Dzudie, A., M Jingi, A., Njume, D., Teuwafeu, D., ... & Kingue, S. (2020). Epidemiology of hypertensive crisis in the Buea Regional Hospital, Cameroon. *The Journal of Clinical Hypertension*, *22*(11), 2105-2110.	Similar cohort with reference number 23

Table S3. Risk of bias.

Study	Design	External validity				Internal validity					Total	Risk of bias
		Was the study's target population a close representation of the national population in relation to relevant variables?	Was the sampling frame a true or close representation of the target population?	Was some form of random selection used to select the sample OR was a census undertaken?	Was the likelihood of nonresponse bias minimal?	Were data collected directly from the subjects (as opposed to a proxy)?	Was an acceptable case definition used in the study?	Was the study instrument that measured the parameter of interest shown to have validity and reliability?	Was the same mode of data collection used for all subjects?	Were the numerator(s) and denominator(s) for the parameter of interest appropriate?		
Rubin et al [32]	Prospective	1	1	1	0	1	1	1	0	1	7	Mod
Gonzalez Pacheco et al [31]	Prospective	0	0	1	0	1	1	1	1	1	6	Mod
Guiga et al [9]	Cross-sectional	1	1	1	0	1	0	1	1	1	7	Mod
Salvetti et al [20]	Prospective	0	1	1	0	1	1	1	1	1	7	Mod
Pinna et al [10]	Cross-sectional	1	1	1	0	1	1	1	1	1	8	Low
Kotruchin et al [24]	Retrospective	1	1	1	0	1	1	1	1	1	8	Low
Vilela-Martin et al [30]	Cross-sectional	1	1	1	0	1	0	1	1	1	7	Mod
Acosta et al [21]	Retrospective	1	1	1	0	0	1	1	0	1	6	Mod
Zampaglione et al [23]	Cross-sectional	1	1	1	0	1	1	1	1	1	8	Low
Martin et al [29]	Retrospective	1	1	1	0	0	0	1	1	1	6	Mod
TajEldin M et al [22]	Cross-sectional	0	0	0	0	1	1	1	1	1	5	High
Pattanshety et al [16]	Retrospective	0	0	1	0	0	1	1	1	1	5	High
Nkoke et al [28]	Cross-sectional	0	1	1	0	1	1	1	1	1	7	Mod

Kim et al [17]	Retrospective	1	1	1	0	1	1	1	1	1	8	Low
Fragoulis et al [27]	Prospective	0	1	1	0	1	1	1	1	1	7	Mod
Benenson et al [26]	Retrospective	0	0	1	0	1	1	1	1	1	6	Mod
Katz et al [25]	Cross-sectional	1	1	1	0	1	0	1	1	1	7	Mod

Mod=Moderate
